# Editorial: Epidemiology and Control of Hepatitis Viruses

**DOI:** 10.3390/life14111369

**Published:** 2024-10-24

**Authors:** Elitsa Golkocheva-Markova

**Affiliations:** NRL Hepatitis Viruses, Virology Department, National Center of Infectious and Parasitic Diseases, 1504 Sofia, Bulgaria; elmarkova2007@gmail.com

Five hepatitis viruses—hepatitis A (HAV), hepatitis B (HBV), hepatitis C (HCV), hepatitis D (HDV), and hepatitis E (HEV)—have a huge impact on human health with their ability to cause acute and often chronic infection. HAV and HEV are transmitted mainly by contaminated water and food, and HBV and HCV are bloodborne; however, all of them can share common transmission routes, such as close contact within a household, blood transfusion, transplantation of an infected allograft, vertical transmission from a pregnant woman to her child, sex with an infected person, and injection drug use. Vaccines exist for HAV, HBV, and HEV, a very efficient treatment is in place for HCV, and a viral control treatment is available for HBV and HDV. To meet the goals set by the World Health Organization for the elimination of viral hepatitis by 2030, it is essential that access to testing is enhanced substantially and novel diagnostic approaches are developed to facilitate and streamline linkage to care. This involves the development of inexpensive diagnostic tools that are administered at or near point-of-care settings. Another important part of the elimination and transmission control strategies is the improved understanding and identification of transmission networks in high-risk populations and communities, as well as the opportunities this provides for efficient use of limited resources, by targeting parts of these networks that would have an optimal impact on the outcome. This Special Issue, “Epidemiology and Control of Hepatitis Viruses” is focused on unique epidemiological patterns, routes of transmission, and opportunities for control of hepatitis viruses. Understanding the global prevalence of these viruses is crucial for effective prevention and treatment strategies.

An estimated one in three individuals in U.S. prisons are infected with HCV, and thus the incarcerated population represents a critical group in the fight to eliminate the virus [1]. Many correctional facilities face challenges in providing consistent and comprehensive HCV care during and after incarceration. While DAAs are available, the number of incarcerated individuals receiving treatment remains low, primarily due to cost concerns, lack of routine screening, and logistical challenges in ensuring continuity of care after release. In their work, Kamat et al. [2] address specifically the continuity of HCV care after an individual is released from incarceration. Former inmates often face housing insecurity and unemployment and hence may lack consistent access to healthcare services and insurance. Individuals leaving prison often experience delays in reactivating their Medicaid coverage, leading to gaps in treatment, as there is often little coordination between prison healthcare services and community-based providers. Addressing these social determinants of health is critical for supporting successful HCV treatment and long-term health outcomes. By expanding access to screening, treatment, and post-release care coordination, states can take important steps toward ensuring that this vulnerable population receives the care they need, ultimately reducing the burden of HCV across the state and beyond.

As evidenced by the data, those at risk are the most affected by the challenges associated with hepatitis elimination. The recent review by Tsaneva-Damyanova and Georgieva, “Epidemiology Pattern, Prevalent Genotype Distribution, Fighting Stigma, and Control Options for Hepatitis D in Bulgaria and Other European Countries” [3], highlights the persistent challenge HDV poses in Bulgaria and other European nations. Despite global efforts to reduce HBV and HDV infections, chronic hepatitis D remains a significant health burden, particularly among vulnerable populations like immigrants from endemic regions. One of the primary concerns discussed is the increased prevalence of chronic HDV in several European countries. Although vaccination programs have decreased HBV infection rates, HDV persists, particularly in immigrant communities from regions such as Eastern Europe, Africa, and Turkey. In Bulgaria, an estimated 10.6% of prison inmates were positive for HDV antibodies, illustrating the virus’s prevalence in high-risk groups. The review emphasizes that, despite a decrease in domestic infections, HDV remains a growing problem, driven by migration and the virus’s ability to rapidly accelerate liver disease progression compared to HBV alone. A major highlight of the review is the limited access to therapeutic options for HDV. Current therapeutic strategies (pegylated interferon) have limited efficacy, while new therapies, such as Bulevirtide (Hepcludex), remain available primarily in Europe. Beyond the medical challenges, the social stigma attached to hepatitis D further complicates management. Up to 90% of chronic HDV patients in Bulgaria experiencing discrimination, not only from society but also from healthcare providers. Stigma limits access to quality healthcare and affects the mental well-being, exacerbating the overall burden of the disease. The findings from Bulgaria show that, without targeted national programs for screening, prevention, and management of chronic viral hepatitis, these efforts will remain insufficient. It is critical for Bulgaria and other European countries to intensify their public health campaigns, increase awareness about HDV, and invest in research for more effective treatment solutions

As antiviral therapies remain limited, especially for HDV, research efforts have shifted toward developing more effective cell culture systems to study these viruses. The review by Lee, Purdy, and Choi in Life [4] addresses the need for robust in vitro models that support the complete life cycle of HBV and HDV infections. These models are also essential for developing curative treatments. Current treatments for HBV, such as nucleoside analogs, suppress viral replication but do not eliminate HBV from infected cells, primarily due to the persistence of covalently closed circular DNA (cccDNA). The review highlights the importance of advanced cell culture systems in identifying drug targets and testing antiviral agents. Systems like HepaRG, Huh7, and HepG2-NTCP cells offer significant improvements over traditional models, especially with the introduction of the sodium taurocholate co-transporting polypeptide receptor (NTCP)-expressing cell lines. NTCP is vital for HBV and HDV entry into hepatocytes, making these modified cells powerful tools for studying viral entry, replication, and pathogenesis. The development of HepG2-NTCP sec+ and the self-assembling co-cultured primary human hepatocytes (SACC-PHHs) represents a breakthrough, enabling the long-term study of both HBV and HDV infections. These systems overcome many limitations of the previous models, such as short infection periods and poor viral replication, and offer more accurate in vivo representation. The integration of three-dimensional (3D) culture models also opens new possibilities for maintaining liver-specific functions and studying the viruses in a more physiologically relevant environment. Challenges remain, such as infection efficiency often requiring external factors like polyethylene glycol (PEG) and dimethyl sulfoxide (DMSO) to enhance viral entry and replication, or the limited availability and lifespan of primary human hepatocytes (PHHs).

The retrospective observational study conducted in Romania within the pediatric population of Romania [5] sheds light on the complex dynamics of pediatric HBV in an under-researched population. This study, the first of its kind in Romania, covers an extended period (1982–2023) and provides invaluable epidemiological data on 506 pediatric patients with chronic HBV infection, offering a robust analysis of their clinical, biochemical, and virological profiles. A notable finding from the study is that nearly 65.4% of the pediatric patients were in the immune-active state, highlighting the need for early diagnosis and treatment to prevent long-term complications such as liver cirrhosis or hepatocellular carcinoma. An alarming observation is the significant prevalence of HBV-HDV coinfections, present in 11.2% of this pediatric cohort, which is a cause for concern due to the more severe clinical progression of hepatitis D co-infections. This percentage, while lower than in the general Romanian population, is still high for a pediatric demographic. HBV-HCV coinfections were rarer, affecting only 1% of the children, a statistic in line with global trends. The correlation between viral load and liver damage validates the need for routine monitoring in pediatric HBV patients. The research demonstrates that, despite concerted vaccination efforts, HBV cases did not decrease significantly in the first decade after the introduction of the HBV vaccine, and Romania continues to grapple with high HBV infection rates. Such data signal gaps in public health strategies, particularly in ensuring complete immunization coverage and effective mother-to-child transmission prevention protocols. In a country with intermediate to high HBV prevalence, these findings should spur policymakers and healthcare professionals into action to improve screening and ensure that pediatric patients with chronic HBV receive timely treatment.

Hepatitis E virus (HEV) has traditionally been associated with poor sanitation and water quality, but in recent years, it has emerged as a significant pathogen in developed countries. HEV in Bulgaria is transmitted mainly through zoonotic pathways such as the consumption of undercooked pork or contact with infected animals. The recent study by Golkocheva-Markova et al. [6] provides valuable insights into age and gender trends in HEV exposure and offers critical implications for public health strategies. It highlights a notable overall seroprevalence of past HEV infections of 10.6%, differing significantly across population sub-groups (5.9–24.5%). Recent or ongoing HEV cases are found in 7.5% of the population, ranging between 2.1% and 20.4% across sub-populations. These findings reveal that HEV remains a significant public health issue in Bulgaria, particularly in high-risk groups such as patients with Guillain–Barré syndrome (GBS) and individuals with underlying liver conditions. The study highlights a clear age-related pattern in HEV seroprevalence, where older adults exhibited a higher prevalence of past HEV infection. This trend is consistent with studies across Europe, suggesting that cumulative exposure to HEV over time leads to higher seroprevalence in older populations. In Bulgaria, this age effect was especially pronounced in sub-populations such as blood donors and patients with chronic liver conditions. Younger individuals were more likely to present markers of current or recent HEV infection, while older age groups had lower rates of such markers, suggesting that younger individuals are at higher risk of contracting HEV due to occupational exposure or dietary habits. The study identified distinct gender differences in HEV exposure, with men exhibiting a higher prevalence of both past and recent/ongoing HEV, nearly double the rate for women. This gender disparity was particularly pronounced in sub-populations, such as blood donors and patients with GBS, and mirrors global trends, where men have more frequent occupational exposure to HEV (e.g., farming or livestock handling) or dietary habits including the consumption of undercooked meat. The study also found that, in certain sub-populations, such as underlying liver conditions or HIV, women exhibited a higher prevalence of past HEV infection, an indication that gender differences in HEV exposure may be context-specific, influenced by health and lifestyle factors. The study emphasizes the need for targeted public health interventions, particularly for high-risk groups such as patients with GBS, chronic liver conditions, and undergoing hemodialysis, who were also found to be at increased risk for HEV exposure. These findings underline the importance of routine HEV screening in vulnerable populations and organizing of public health awareness campaigns focusing on HEV prevention.

The potential for transmission of the hepatitis E virus from animals to humans is well documented despite the absence of direct evidence of such transmission. The hypothesis is typically based on the established close genetic relationship between isolates of the hepatitis E virus in animals and humans [7]. The study, conducted by Tene et al. [8] presents groundbreaking research on the detection of the Hepatitis E Virus (HEV) in pork sold in Saint-Louis, Senegal. This work represents the first investigation into HEV contamination in pork products intended for human consumption in the region and underlines the importance of assessing zoonotic risks associated with pork products. With 5.4% of pork samples testing positive for HEV RNA, the findings indicate a potential health risk, particularly due to the high contamination rate of pork liver (22.2%). In Senegal, where pork consumption is relatively low and confined to specific communities, the study raises awareness about the risk of HEV transmission through poorly regulated meat markets. The results call for the implementation of strict meat inspection and control measures to ensure food safety from farm to fork. Furthermore, the study advocates for a One Health approach, incorporating environmental, animal, and human health perspectives to comprehensively address zoonotic threats like HEV. The authors highlight the need for a coordinated response involving food safety authorities, public health agencies, and the research community to protect consumers and prevent the spread of HEV from animals to humans.

This Special Issue brings together viral hepatitis researchers from around the world. The knowledge provided can assist in developing effective public health strategies to control viral hepatitis worldwide by understanding the life cycle and prevalence patterns of hepatitis viruses, improving screening efforts, targeting at-risk populations, adapting to changing local and global epidemiological trends, and filling existing gaps in the One Health approach.

## References

[1] Hoff E., Warden A., Taylor R., Nijhawan A.E. (2023). Hepatitis C Epidemiology in a Large Urban Jail: A Changing Demographic. Public Health Rep..

[2] Kamat S., Kondapalli S., Syed S., Price G., Danias G., Gorbenko K., Cantor J., Valera P., Shah A.K., Akiyama M.J. (2023). Access to Hepatitis C Treatment During and After Incarceration in New Jersey, United States: A Qualitative Study. Life.

[3] Tsaneva-Damyanova D.T., Georgieva L.H. (2023). Epidemiology Pattern, Prevalent Genotype Distribution, Fighting Stigma and Control Options for Hepatitis D in Bulgaria and Other European Countries. Life.

[4] Lee G.S., Purdy M.A., Choi Y. (2023). Cell Culture Systems for Studying Hepatitis B and Hepatitis D Virus Infections. Life.

[5] Păcurar D., Dinulescu A., Jugulete G., Păsărică A.-S., Dijmărescu I. (2024). Hepatitis B in Pediatric Population: Observational Retrospective Study in Romania. Life.

[6] Golkocheva-Markova E., Ismailova C., Kevorkyan A., Raycheva R., Zhelyazkova S., Kotsev S., Pishmisheva M., Rangelova V., Stoyanova A., Yoncheva V. (2023). Age and Gender Trends in the Prevalence of Markers for Hepatitis E Virus Exposure in the Heterogeneous Bulgarian Population. Life.

[7] Zheng Y., Ge S., Zhang J., Guo Q., Ng M.H., Wang F., Xia N., Jiang Q. (2006). Swine as a Principal Reservoir of Hepatitis E Virus That Infects Humans in Eastern China. J. Infect. Diseases.

[8] Tene S.D., Diouara A.A.M., Kane A., Sané S., Coundoul S., Thiam F., Nguer C.M., Diop M., Mbaye M.N., Mbengue M. (2024). Detection of Hepatitis E Virus (HEV) in Pork Sold in Saint-Louis, the North of Senegal. Life.

