# Correction: Golkocheva-Markova, E. Editorial: Epidemiology and Control of Hepatitis Viruses. *Life* 2024, *14*, 1369

**DOI:** 10.3390/life15050711

**Published:** 2025-04-28

**Authors:** Elitsa Golkocheva-Markova

**Affiliations:** NRL Hepatitis Viruses, Virology Department, National Center of Infectious and Parasitic Diseases, 1504 Sofia, Bulgaria; elmarkova2007@gmail.com

Following a request from the scientific organization with which she is affiliated, the second author wishes to remove her name from the author collective in previous publication [1].

The author states that the scientific conclusions are unaffected. This correction was approved by the Academic Editor. The original publication has also been updated.
